# Diaphragm Ultrasonography: Reference Values and Influencing Factors for Thickness, Thickening Fraction, and Excursion in the Seated Position

**DOI:** 10.1007/s00408-023-00662-2

**Published:** 2023-11-29

**Authors:** Toru Yamada, Taro Minami, Syumpei Yoshino, Ken Emoto, Suguru Mabuchi, Ryoichi Hanazawa, Akihiro Hirakawa, Masayoshi Hashimoto

**Affiliations:** 1 Department of General Medicine, Graduate School of Medical and Dental Sciences, Tokyo Medical and Dental University, Bunkyo-ku, Tokyo, 113-8510 Japan; 2 Medicine, Division of Pulmonary, Critical Care, and Sleep Medicine, The Warren Alpert Medical School of Brown University, Providence, RI 02903 USA; 3 General Internal Medicine, Iizuka Hospital, Iizuka, 135-0041 Fukuoka Japan; 4 General Internal Medicine, Kaita Hospital, Iizuka, 820-1114 Fukuoka Japan; 5 Department of Clinical Biostatistics, Graduate School of Medical and Dental Sciences, Tokyo Medical and Dental University, Bunkyo-ku, Tokyo, 113-8510 Japan

**Keywords:** Diaphragm, Reference Values, Seated Position, Ultrasonography

## Abstract

**Introduction:**

Measurements of diaphragm function by ultrasonography are affected by body position, but reference values in the seated position have not been established for an Asian population. This study aimed to determine reference values for diaphragm thickness, thickening fraction, and dome excursion by ultrasonography and to investigate the effects of sex, height, and body mass index.

**Methods:**

Diaphragm ultrasonography was performed on 109 seated Japanese volunteers with normal respiratory function who were enrolled between March 2022 and January 2023. Thickness, thickening fraction, and excursion were measured. Reference values and the measurement success rate were calculated. Multivariate analysis adjusted for sex, height, and body mass index was performed.

**Results:**

The measurement success rate was better for thickness than for excursion. The mean (lower limit of normal) values on the right/left sides were as follows. During quiet breathing, thickness at end expiration(mm) was 1.7 (0.9)/1.6 (0.80), thickening fraction(%) was 50 (0.0)/52 (0.0), and excursion(cm) was 1.7 (0.5)/1.9 (0.5). During deep breathing, the thickening fraction was 111 (24)/107 (22), and the excursion was 4.4 (1.7)/4.1 (2.0). In multivariate analysis, body mass index was positively associated with thickness but not with the thickening fraction.

**Conclusion:**

The reference values in this study were smaller than those in previous reports from Europe. Considering that thickness is influenced by body mass index, using Western reference values in Asia, where the average body mass index is lower, might not be appropriate. The thickening fraction in deep breathing is unaffected by other items and can be used more universally.

## Introduction

Bilateral diaphragmatic dysfunction leads to symptoms such as dyspnea, chest pain, and sleep disturbance [1, 2]. Symptoms are influenced by body position, worsening in a supine position and improving when seated [1–3]. Diaphragmatic dysfunction may be caused by ventilator-induced injury, surgery, and respiratory, neurological, musculoskeletal, and neuromuscular junction diseases [2–5]. Ultrasonography has a sensitivity and specificity of more than 90% for diagnosis of diaphragmatic dysfunction, [2, 6–8] but is not a widely accepted assessment method. One potential cause for underutilization is that testing methods and reference values are not well established [2, 6, 8].

Two ultrasonographic methods can be used to measure diaphragmatic function: (1) measurement of diaphragmatic excursion during breathing and (2) measurement of the thickness of the diaphragm (Tdi) and its rate of change (i.e., the thickening fraction [TF]) during breathing. Both excursion and TF are influenced by body position. Excursion is greater in the supine position than in the seated position, whereas TF is greater in the seated position [8–11]. Therefore, there is a need to establish reference values according to the body position in which measurements are obtained. Most published reports refer to measurements obtained in the supine or semi-recumbent position, and studies of seated reference Tdi, TF, and excursion values are scarce [1, 8, 12]. In the outpatient setting, it is easier to obtain measurements in the seated position, which is preferred by patients with diaphragmatic dysfunction because of orthopnea. Establishing reference values in the seated position is important in promoting the use of diaphragm ultrasonography. Furthermore, most reports on reference values are based on Western populations, which may not be appropriate for Asians, who generally have a different physical build. The only relevant reports involving Asians are based on healthy populations with sample sizes of fewer than 30 individuals or patients with a history of stroke or spinal cord injury [13–16]. There has not been a large-scale report on reference values for healthy Asians.

This study aimed to determine reference values for Tdi, TF, and excursion of the diaphragm dome obtained by ultrasonography in healthy seated Japanese volunteers and to investigate the effects of sex, height, and body mass index (BMI) on the measurements obtained.

## Methods

### Study Population and Setting

Healthy adult volunteers were recruited from Tokyo Medical and Dental University Hospital (Tokyo), Iizuka Hospital (Fukuoka), and Shinai Clinic (Kanagawa), between March 2022 and January 2023 in Japan. General information such as age, sex, height, weight, and medical history was collected on the day of the examination. Respiratory function tests were performed using a spirometer (AutoSpiro507; Minato Medical Science Co., Ltd, Osaka, Japan). Volunteers with no symptoms, a percent vital capacity ≥ 80, and a forced expiratory volume in one second (FEV_1_)/forced vital capacity (FVC) of ≥ 70% were included. All measurements were obtained by ultrasonography technicians or physicians who were certified as instructors in point-of-care ultrasound simulation courses [17].

### Thickness, Thickening Fraction, and Excursion

**Fig. 1:**
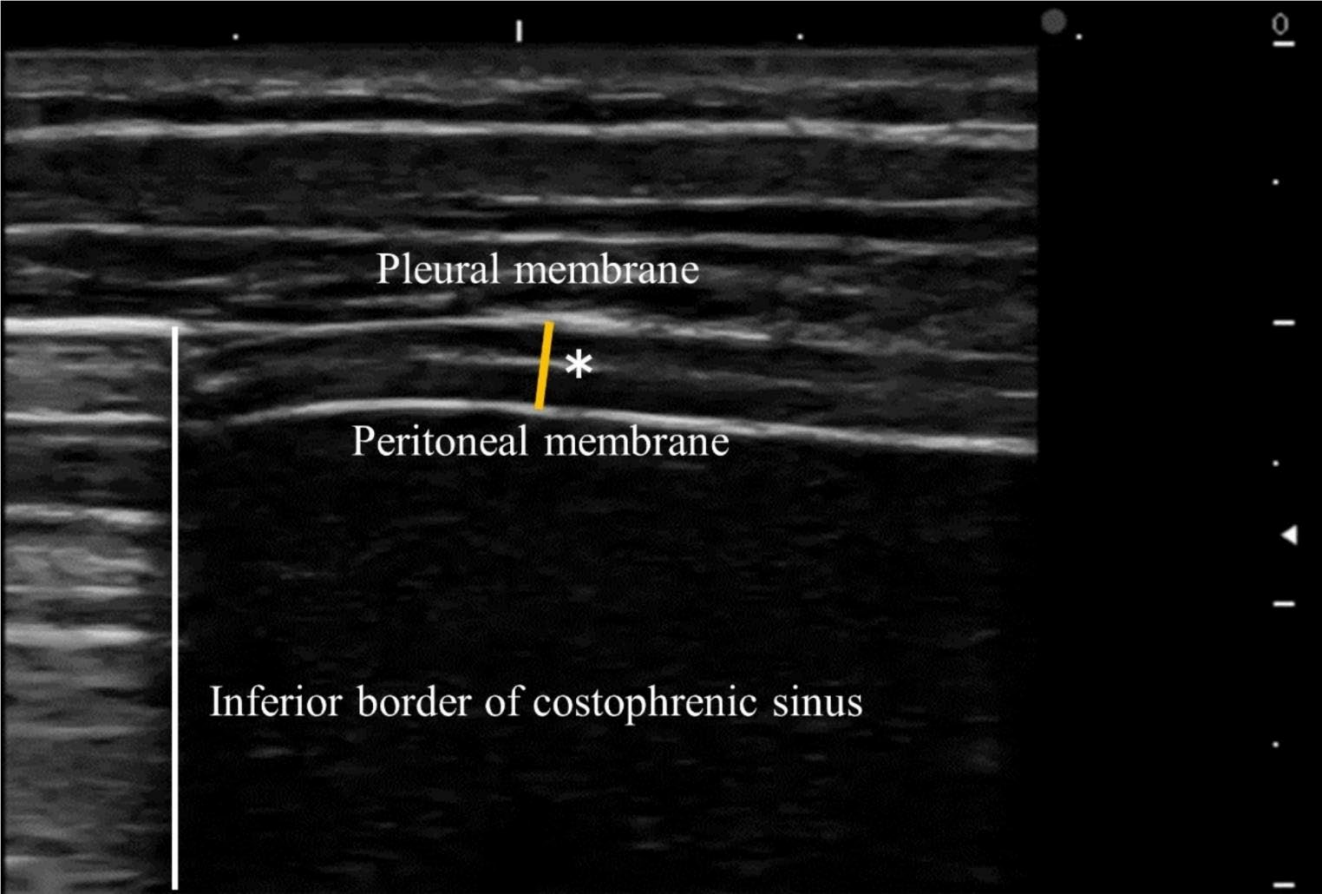
Measurement of diaphragm thickness. The diaphragm was observed as a three-layer structure, consisting of a hypoechoic muscular layer sandwiched between two bright white lines (the pleural and peritoneal membranes), with a white line running in the center (considered to be the fibrous layer in the center of the diaphragm). *Measured in B-mode by placing a measurement marker from the center of the thickness of the pleural membrane to the center of the thickness of the peritoneal membrane

In the seated position, Tdi was measured using a linear transducer (7.0 MHz) placed vertically around the eighth to ninth intercostal space, aligned perpendicular to the chest, avoiding ribs. The height was set to a position where a portion of the lung could be observed at the edge of the screen during inspiration. Tdi was measured at end expiration and end-inspiration during quiet breathing (QB) and deep breathing (DB). The method is depicted in Fig. 1. TF was calculated using the following formula:

end-inspiration Tdi (total lung capacity [TLC]) – end-expiration Tdi (functional residual capacity [FRC])/end-expiration Tdi (FRC) × 100.

**Fig. 2:**
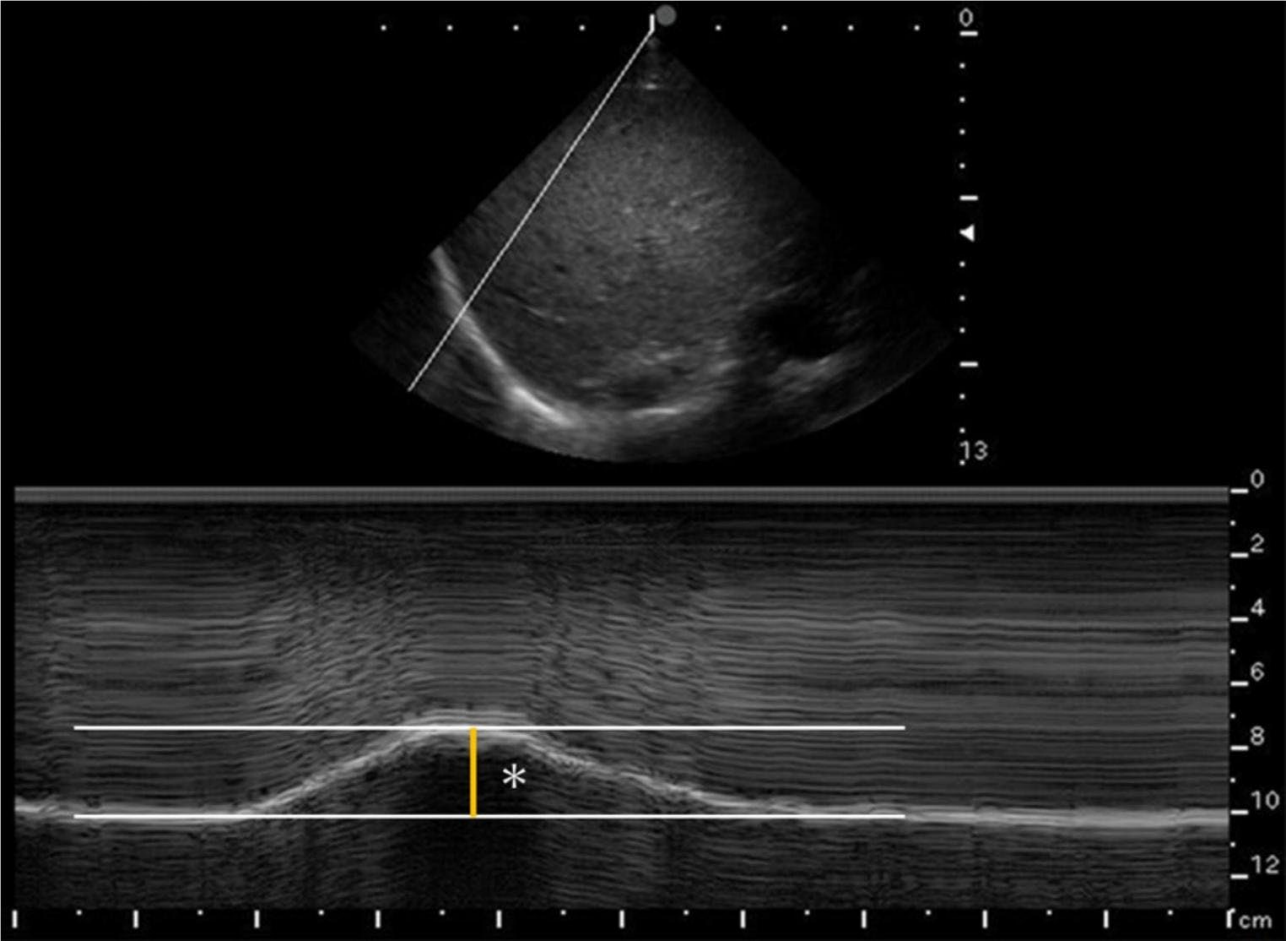
Measurement of diaphragmatic excursion. *Measured in M-mode by placing a measurement line as perpendicular as possible across the diaphragm and measuring changes during inspiration and expiration

Excursion was measured on both sides during QB and DB. The phased-array transducer (2.5 MHz) was placed vertically around the eighth to ninth intercostal space, aligned perpendicular to the chest, avoiding ribs. The M-mode line was adjusted to be as perpendicular to the dome as possible, and its excursion during inspiration was measured. (Fig. 2).

One examiner measured all sites for each subject. Tdi measurement succeeded if both TLC and FRC values were obtained in one breath. Dome excursion was successful if diaphragm movement was shown in one respiratory cycle.

### Statistical Analysis

Subject characteristics are shown as mean and standard deviation for continuous data, and frequency and percentage for categories. The lower limit of normal (LLN) and upper limit of normal (ULN) were calculated as mean ± 1.95 standard deviations. Diaphragm measurements’ left-right and sex differences were compared using paired and two-sample t-tests, respectively. Multiple regressions assessed associations with sex, height, and BMI. Analyses were done using STATA 17.0 (StataCorp LLC, College Station, TX, USA). A p-value < 0.05 was considered statistically significant.

## Results

### Study Participants

A total of 111 Japanese volunteers were recruited during the 10-month study period. Two subjects in whom respiratory function tests revealed a percent vital capacity < 80% were excluded. The remaining 109 subjects were included in the analysis. In terms of history of respiratory or neuromuscular disease, there were two cases of bronchial asthma, but neither showed any symptoms of asthma on the day of examination; both had normal FEV_1_/FVC and percent vital capacity values. The subject characteristics are shown in Table 1.

**Table 1:** Participant characteristics according to sex

	Men	(n = 56, 51%)	Women	(n = 53, 49%)		
Age (years)	30.3	(SD 8.9)	33.5	(SD 13.3)		
Height (cm)	172.2	(SD 6.1)	158	(SD 6.1)		
Body mass index	22.5	(SD 2.9)	21.7	(SD 2.6)		
%VC	97.8	(SD 9.3)	95.1	(SD 10.7)		
FEV_1_/FVC	85.2	(SD 4.7)	86	(SD 5.6)		
Smoking history						
Never smoker	49	(87.5%)	45	(84.9%)		
Former smoker	5	(8.9%)	7	(13.2%)		
Current smoker	2	(3.6%)	1	(1.9%)		
Past medical history						
Asthma	1	(1.8%)	1	(1.9%)		

%VC, percent vital capacity; FEV_1_/FVC, forced expiratory volume in one second/forced vital capacity; SD, standard deviation

## Measurement Success rate

Tdi was measurable in all cases with a 100% success rate. The success rate of dome excursion was generally lower on the left, especially during DB, mainly because the diaphragm was obscured by the lung. Impaired observation owing to fat attenuation was not a significant issue. Table 2 shows the success rates at each measurement site and the reasons for measurement failure.

**Table 2:** Success rate according to ultrasonographic parameter measured

Parameter	Respiratory pattern	Right/Left hemidiaphragm	Success rate (n)	Failures with reason
Diaphragm thickness	Quiet breathing	Right	100% (109/109)	―
		Left	100% (109/109)	―
	Deep breathing	Right	100% (109/109)	―
		Left	100% (109/109)	―
Diaphragm excursion	Quiet breathing	Right	99.0% (108/109)	Lung, n = 1
		Left	92.7% (101/109)	Lung, n = 5; Stomach, n = 2; Fat, n = 1
	Deep breathing	Right	90.8% (99/109)	Lung, n = 10
		Left	45.9% (50/109)	Lung, n = 54; Stomach, n = 4; Fat, n = 1

Lung: the diaphragm is hidden by the lungs during inspiration. Stomach: the diaphragm is hidden by the stomach. Fat: the diaphragm cannot be observed because of attenuation by fat. Heart: the diaphragm is hidden by the heart

### Thickness, Thickening Fraction, and Excursion

Mean Tdi at functional residual capacity (FRC) was significantly thicker on the right side than on the left side. Furthermore, both sides were significantly thicker in men than in women. There was no significant difference in TF between the left and right sides, nor any significant sex-related differences. There was a statistically significant difference in diaphragmatic excursion between the left and right sides during QB but not during DB. Movement of the diaphragm was greater in men during DB. The mean, LLN, and ULN values for each parameter are presented in Tables 3 and 4.

**Table 3:** Normal values for diaphragm thickness at end-expiration and thickening fraction

Parameter	Right/Left hemidiaphragm	Mean (SD), mm						LLN, ULN, mm		
		Total	p-value*	Men	Women	p-value^†^		Total	Men	Women
Thickness at end-expiration (FRC)	Right (n = 109)	1.7 (0.4)	< 0.01	1.8 (0.3)	1.6 (0.5)	0.03		0.9, 2.5	1.2, 2.4	0.6, 2.6
	Left (n = 109)	1.6 (0.4)		1.7 (0.4)	1.5 (0.4)	0.03		0.8, 2.4	0.9, 2.5	0.7, 2.3
		Mean (SD), %						LLN, ULN, %^‡^		
		Total	p-value*	Men	Women	p-value^†^		Total	Men	Women
Thickening fraction (Quiet breathing)	Right (n = 109)	50.0 (25.9)	0.59	46.4 (20.8)	53.8 (30.0)	0.14		0.0, 100.5	5.8, 87.0	0.0, 112.3
	Left (n = 109)	51.8 (26.9)		49.5 (23.6)	54.2 (30.0)	0.36		0.0, 104.3	3.5, 95.5	0.0, 112.7
Thickening fraction (Deep breathing)	Right (n = 109)	110.7 (44.3)	0.50	113.8 (46.0)	107.4 (42.6)	0.45		24.3, 197.1	24.1, 203.5	24.3, 190.5
	Left (n = 109)	107.2 (43.8)		108.6 (42.7)	105.6 (45.4)	0.72		21.8, 192.6	25.3, 191.9	17.1, 194.1

FRC, functional residual capacity; LLN, lower limit of normal; SD, standard deviation; ULN, upper limit of normal. *p-value for right vs. left. ^†^p-value for men vs. women. ^‡^If LLN was less than 0, it was set to 0

**Table 4:** Normal values for diaphragm excursion

Parameter	Respiratory pattern	Right/Left hemidiaphragm	Mean (SD), cm						LLN, ULN, cm^‡^		
			Total	p-value*	Men	Women	p-value^†^		Total	Men	Women
Diaphragm excursion	Quiet breathing	Right (n = 108)	1.7 (0.6)	0.04	1.8 (0.6)	1.6 (0.6)	0.05		0.5, 2.9	0.6, 3.0	0.4, 2.8
		Left (n = 101)	1.9 (0.7)		2.0 (0.7)	1.7 (0.6)	0.11		0.5, 3.3	0.6, 3.4	0.5, 2.9
	Deep breathing	Right (n = 99)	4.4 (1.4)	0.83	5.1 (1.3)	3.8 (1.1)	< 0.01		1.7, 7.1	2.6, 7.6	1.7, 6.0
		Left (n = 50)	4.1 (1.1)		4.7 (1.1)	3.8 (1.0)	< 0.01		2.0, 6.3	2.6, 6.9	1.9, 5.8

LLN, lower limit of normal; SD, standard deviation; ULN, upper limit of normal. *p-value for right vs. left. ^†^p-value for men vs. women. ^‡^If LLN was less than 0, it was set to 0

### Multiple Linear Regression Analyses

Multiple linear regression analyses were performed with adjustment for sex, height, and BMI. Significant associations of BMI with left and right Tdi, TF of the left diaphragm during QB, and excursion of the right diaphragm during DB were observed. In DB, a correlation was observed between the right excursion and both gender and BMI, but no correlation was found for TF with gender, height, or BMI. All results of analysis are shown in Table 5.

**Table 5:** Results of multiple regression analysis adjusted for sex, height, and body mass index

Parameter	Respiratory pattern	Right/Left hemidiaphragm	Female sex (reference, male)		Height		Body mass index	
			Regression coefficient (95% CI)	p-value	Regression coefficient (95% CI)	p-value	Regression coefficient (95% CI)	p-value
Thickness at end-expiration	FRC	Right (n = 109)	-0.2 (-0.42, 0.04)	0.11	0.0 (-0.2, 0.01)	0.52	0.1 (0.03, 0.09)	< 0.01*
		Left (n = 109)	0.0 (-0.18, 0.20)	0.92	0.0 (<-0.01, 0.02)	0.13	0.1 (0.05, 0.09)	< 0.01*
Thickening fraction	Quiet breathing	Right (n = 109)	12.3 (-2.98, 27.67)	0.11	0.3 (-0.49, 1.15)	0.43	0.5 (-1.35, 2.26)	0.62
		Left (n = 109)	-1.2 (-16.89, 14.53)	0.88	-0.3 (-1.14, 0.53)	0.48	-2.0 (-3.87, -0.17)	0.03*
	Deep breathing	Right (n = 109)	-5.1 (-31.62, 21.45)	0.71	0.1 (-1.31, 1.52)	0.88	-0.3 (-3.39, 2.85)	0.86
		Left (n = 109)	7.3 (-18.66, 33.34)	0.58	0.8 (-0.56, 2.21)	0.24	-1.7 (-4.76, 1.36)	0.27
Diaphragm excursion	Quiet breathing	Right (n = 108)	-0.8 (-4.30, 2.74)	0.66	0.1 (-0.08, 0.29)	0.28	0.0 (-0.37, 0.47)	0.82
		Left (n = 101)	-3.7 (-7.60, 0.27)	0.07	-0.1 (-0.34, 0.08)	0.22	0.5 (-0.05, 0.96)	0.07
	Deep breathing	Right (n = 99)	-11.8 (-19.20, -4.43)	< 0.01*	0.0 (-0.35, 0.44)	0.82	1.0 (0.09, 1.94)	0.03*
		Left (n = 50)	-9.7 (-19.54, 0.10)	0.05	-0.1 (-0.54, 0.38)	0.72	-0.1 (-1.46, 1.24)	0.87

CI, confidence interval; FRC, functional residual capacity. *p < 0.05

## Discussion

This study determined seated reference values for Tdi, TF, and excursion in 109 healthy Japanese individuals. This is the largest-ever study to include simultaneous measurement of Tdi, TF, and excursion in seated Asian subjects and provides a benchmark for normal seated values in the Asian population.

The success rate for measurement of Tdi and TF was 100% regardless of side or breathing pattern. The main problem in measuring excursion was the overlapping of the lungs during inspiration, but fat attenuation was not a significant problem. Although diaphragm ultrasonography is more helpful in obtaining measurements during DB than during QB, [8] the success rate for excursion in DB was lower than that for Tdi and TF measurements. Therefore, measurement of TF, which has a higher success rate during DB and is less influenced by sex, height, and BMI, might be more useful than measurement of excursion.

Previous reports have indicated that it is easier to measure excursion than TF [6]. However, our study yielded different results. This inconsistency might be explained by differences in BMI between studies. The mean BMI in our study was 22.1, and the percentage of subjects with BMI ≥ 30 was 1%. Given that subcutaneous fat can hinder visibility with the linear transducer used for measuring TF, our high success rate when measuring Tdi may reflect a low obesity rate. The average BMI varies markedly from country to country. For example, the average adult BMI in Japan is 23.6 for men and 21.8 for women, 28.8 and 28.9 in the US, and 27.3 and 27.0 in the UK [18]. In Japan, only 5.4% of men and 3.6% of women have a BMI ≥ 30; these rates are considerably lower than those in Western countries [18, 19]. In populations where obesity is not a significant issue, as in our study, it might be advisable to first consider measuring the TF.

Past reports of Tdi at FRC range from 1.6 to 3.8 mm in men and 1.4 to 2.7 mm in women [1, 8, 13, 14, 20–24]. The results of the present study are consistent with previous reports. Our results for the LLN in men are close to those of Cardenas et al. and Boussuges et al.; however, the mean Tdi in women was more than 40% less than in the earlier studies [1, 20]. One of the criteria for a diagnosis of diaphragmatic weakness is a Tdi of < 2 mm at end expiration [2, 8, 25]; however, considering past reports and our present findings, many healthy individuals fall below this standard, suggesting that this criterion may be inappropriate for diagnosing diaphragmatic paralysis.

Our finding that the diaphragm was significantly thicker in men than in women is consistent with past reports [1, 8, 24, 26, 27]. However, in multiple linear regression analysis adjusted for sex, height, and BMI, there was no association with sex (right, p = 0.1; left, p = 0.92), but there was a statistically significant relationship with BMI (right, p < 0.01; left, p < 0.01). Other studies have also reported Tdi at FRC showed positive correlations with BMI [1, 26, 28]. Our results support these findings regarding the correlation with BMI but not with sex. It is unlikely that our failure to find a statistically significant correlation with sex was attributable to an insufficient sample size, given that the size of our study population was adequate when compared with those in the previous studies. While it appears that the diaphragm is generally thicker in men than in women, Tdi may be influenced more by BMI than by sex. As previously mentioned, the average BMI varies widely by country [18]. Considering that Tdi is influenced by BMI, standard values developed in Western countries, where the average BMI is significantly higher than that in Asian countries, may be inappropriate for use in Japan and other Asian populations.

During QB, even healthy individuals may have a change in Tdi of < 10% or no change at all [1, 27]. In our study, the TF was < 20% during QB in two cases (2%) on the right and in six cases (6%) on the left, and there was one case (1%) with a TF < 10% on the left. Therefore, TF in DB is more useful than TF in QB for evaluating diaphragmatic function and diagnosing diaphragm paralysis [1, 27].

TF is markedly affected by body position, with changes increasing in the order of supine, seated, and standing [10, 11]. In studies that examined changes in TF according to body position, the average TF values in DB were 60% in the supine position, 97% in the seated position, and 174% in the standing position [11]. Therefore, our results should be compared with previously reported measurements obtained in the seated position. However, there are limited data on normal seated TF values. In the only large-scale study of seated TF [1], which included 200 healthy individuals, the mean TF (LLN) in DB was 106% (40%)/112% (39%) for men and 116% (39%)/121% (48%) for women, with no apparent sex differences. Our mean values are close to those in the previous report. Although we calculated the LLN in the same way as the previous report [1] (i.e., the mean ± a standard deviation of 1.95), our results were about 20% lower than in their report.

A value of TF should be considered as indicating dysfunction if it is greater than the criterion for complete paralysis (TF < 20%) and less than the LLN [2]. However, a consensus has not yet been reached on the cut-off value for dysfunction [6]. Considering that the LLN was in the 20% range in our study, the original criterion for diaphragmatic paralysis (TF < 20%) was calculated from data obtained in the standing position, [25] and the fact that the seated TF is smaller than the standing TF [11] suggests there may be room for reconsideration of application of a TF < 20% as a criterion for diagnosis of diaphragmatic paralysis when measurements are obtained in the seated position.

Our multiple linear regression analysis adjusted for sex, height, and BMI found no association of the TF in DB with any of these three parameters. Tdi is affected by BMI, so it is difficult to refer to Western values in Asians, whose physique differs from that of Westerners. TF in DB is less affected by BMI and other factors, it may be used as a more generalized value.

Diaphragmatic dome excursion is also affected by body position, but, unlike TF, becomes larger in the supine position than in the seated position [8, 9]. Since abdominal compliance is less when seated, diaphragm dome excursion may be less for a given degree of diaphragm activation or tidal volume. Therefore, our results should be compared with those previously obtained in the seated position. However, there are few relevant reports. In the only large-scale study, Boussuges et al. measured excursion in the seated position and reported that the mean (LLN) values in QB were 1.9 cm (0.9 cm) on the right and 2.0 cm (0.9 cm) on the left in men and 1.7 cm (0.9 cm) and 1.7 cm (0.9 cm), respectively, on the right in women. In DB, the mean (LLN) values were 6.6 cm (4.1 cm) on the right and 6.7 cm (4.2 cm) on the left in men and 5.4 cm (3.3 cm) and 5.4 cm (3.2 cm), respectively, in women [12]. Our findings were equivalent to those of Boussuges et al. in QB, but were 1.5–2.0 cm smaller in DB in both sexes. Moreover, the LLN was 40–60% smaller in both QB and DB for both sexes. Multiple linear regression analysis identified a statistically significant association of excursion with female sex (p < 0.01) and BMI (p = 0.03) on the right side in DB but no association on the left side. The lack of a significant association on the left may reflect the inadequate sample size caused by the low rate of successful depiction. One reason why our excursion values in DB were smaller than those in the study by Boussuges et al. could be that the mean BMI was lower in our study subjects than in those in their study (22.5 vs. 25.0 for men and 21.7 vs. 25.0 for women). [12] It has been proposed that the criterion for diaphragm dysfunction with excursion in QB should be < 2 cm [6]. Considering past findings and our present results and that seated excursion is smaller than supine excursion, [8, 9] another criterion should be considered for the seated position.

This study has several limitations. The success rate for measurement of Tdi was higher than that for excursion, which might reflect the lower obesity rate in our study population. Therefore, it is unclear whether our results are applicable to populations with higher obesity rates. Further study is needed on a wider range of age groups, body size, and races.

In summary, we have determined reference values for use when performing diaphragm echography in a seated position. Considering that Tdi and excursion are influenced by BMI, it may not be possible to directly apply the reference values developed for the West in Asian countries, which have different obesity rates. The TF in DB does not appear to be affected by sex, height, or BMI and could be utilized more widely than other criteria. The current standards for diagnosis of diaphragmatic paralysis may need to be reconsidered for use in a seated position, given that many healthy individuals meet the criteria when measured in this manner.

## Abbreviations

Tdi: thickness of the diaphragm
BMI: body mass index
DB: deep breathing
FEV_1_: forced expiratory volume in one second
FRC: functional residual capacity
FVC: forced vital capacity
LLN: lower limit of normal
QB: quiet breathing
TF: thickening fraction
TLC: total lung capacity
ULN: upper limit of normal
